# Iron and iron-related proteins in COVID-19

**DOI:** 10.1007/s10238-022-00851-y

**Published:** 2022-07-18

**Authors:** Erin Suriawinata, Kosha J. Mehta

**Affiliations:** 1 Faculty of Life Sciences and Medicine, King’s College London, London, UK; 2 Centre for Education, Faculty of Life Sciences and Medicine, King’s College London, London, UK

**Keywords:** COVID, SARS-CoV-2, Iron, Ferritin, Hepcidin, Transferrin

## Abstract

COVID-19 can cause detrimental effects on health. Vaccines have helped in reducing disease severity and transmission but their long-term effects on health and effectiveness against future viral variants remain unknown. COVID-19 pathogenesis involves alteration in iron homeostasis. Thus, a contextual understanding of iron-related parameters would be very valuable for disease prognosis and therapeutics.

Accordingly, we reviewed the status of iron and iron-related proteins in COVID-19. Iron-associated alterations in COVID-19 reported *hitherto* include anemia of inflammation, low levels of serum iron (hypoferremia), transferrin and transferrin saturation, and high levels of serum ferritin (hyperferritinemia), hepcidin, lipocalin-2, catalytic iron, and soluble transferrin receptor (in ICU patients). Hemoglobin levels can be low or normal, and compromised hemoglobin function has been proposed. Membrane-bound transferrin receptor may facilitate viral entry, so it acts as a potential target for antiviral therapy. Lactoferrin can provide natural defense by preventing viral entry and/or inhibiting viral replication. Serum iron and ferritin levels can predict COVID-19-related hospitalization, severity, and mortality. Serum hepcidin and ferritin/transferrin ratio can predict COVID-19 severity. Here, serum levels of these iron-related parameters are provided, caveats of iron chelation for therapy are discussed and the interplay of these iron-related parameters in COVID-19 is explained.

This synopsis is crucial as it clearly presents the iron picture of COVID-19. The information may assist in disease prognosis and/or in formulating iron-related adjunctive strategies that can help reduce infection/inflammation and better manage COVID-19 caused by future variants. Indeed, the current picture will augment as more is revealed about these iron-related parameters in COVID-19.

## Introduction

The Severe Acute Respiratory Syndrome Coronavirus-2 (SARS-CoV-2) causes the Coronavirus disease-2019 (COVID-19). The disease mainly affects the lungs and demonstrates a wide range of clinical spectrum i.e., from asymptomatic infection to mild, moderate, and severe cases, and presents pulmonary dysfunction with arterial hypoxemia, eventually leading to acute respiratory distress syndrome (ARDS) [1]. Severe cases show damage to the epithelial cells in the lungs, thrombosis, hypercoagulation, and vascular leak that causes sepsis. These events culminate in and contribute to ARDS and pulmonary fibrosis [2]. Associated complications include viral pneumonia, respiratory failure, multi-organ dysfunction involving the liver, heart, and kidneys, and mortality [1]. In addition, ischemic strokes, delirium, and seizures are often reported in COVID-19 patients [3].

Indeed, vaccines have helped in reducing disease severity and transmission. However, many aspects remain unknown such as the long-term effect of vaccines on health, the effectiveness of vaccines against future variants, treatment of the severely ill during epidemic waves, and the long-term effect of repeated infections on the health of the vaccinated population. Thus, it is essential to further understand the determinants and regulators of COVID-19 pathology to be able to better tackle future variants and pandemic waves.

Iron is essential for normal cellular and physiological functionality. Low iron levels can lead to anemia, whereas high levels cause excessive oxidative stress that damages cells/organs. Interestingly, both low and high body iron levels increase the risk of infection [4]. Collectively, these aspects show the significance of iron homeostasis in the body. As such, independent of COVID-19, a pattern of high serum ferritin (iron-storage protein) but low serum iron and low transferrin (iron-carrier protein in circulation) within 3 days of ICU admission has been seen in more than 75% of critically ill patients [4], indicating the significance of iron and related proteins in critical illness, and critical illness is seen in COVID-19 cases.

Iron dysregulation is observed in several pathologies [5–8], including respiratory diseases and pulmonary fibrosis that have been linked with higher iron levels in the lungs [9, 10]. A scenario of lower body iron and higher tissue iron has been linked with lower lung function in women aged 20–49 years [11]. Thus, a link between iron and respiratory dysfunction does exist, which suggests a link between iron and COVID-19.

### Link between iron and COVID-19

The two hallmarks of COVID-19 are hyperinflammation and hyperferritinemia [12]. The latter implies elevated levels of the iron-storage protein ferritin. Moreover, alteration of iron homeostasis has been implicated in COVID-19. Iron-related parameters deviated from the norm in SARS-CoV-2-infected outpatients, inpatients and the critically ill patients, where the extent of deviation increased with increasing disease severity [13]. Iron deficiency, and elevations in serum ferritin and hepcidin (iron-hormone) persisted for around 2 months after the onset of COVID-19 in some patients. These iron-related alterations were linked with diminished physical performance and non-resolving lung pathologies [12]. Collectively, these data highlight the involvement of iron and related proteins in COVID-19 pathology.

Whether these alterations are simply bystanders of the disease event or contributors to COVID-19 pathology or a reflection of the physiological response to infection is yet to be fully understood. Analysis of the status and significance of iron and iron-related proteins in COVID-19 may help in a) better understanding disease pathology, b) identifying diagnostic and prognostic markers that could be used in conjunction with the current approaches, and/or c) identifying molecular targets for therapeutic interventions and thereby facilitate disease amelioration.

Accordingly, this review presents a synopsis of the status and interplay of iron and iron-related proteins in COVID-19.

## Overview of iron and iron-related proteins in COVID-19 patients

A study showed that compared to the outpatient cohort, iron-related parameters (serum ferritin, iron, transferrin, and transferrin saturation) were more severely altered in the inpatient cohort and in those outpatients that were later hospitalized due to health deterioration [13].

Table 1 shows the levels of iron and iron-related proteins in COVID-19 patients of varying severities. It includes iron-related data on outpatients that remained home during COVID-19, the inpatients, those in ICU and those with mild, severe, and critical disease.

**Table 1:** Iron and iron-related parameters in COVID-19 patients of varying severities

Iron-related parameter	Reported status in COVID-19	Serum levels [Values in Mean ± SD or Mean ± SEM or Median (Interquartile range), unless otherwise stated]	
		Normal range	In COVID-19 patients
Iron	Low. Can be low even after two months of COVID-19 onset [12]	11–30 μmol/L [17]	COVID-19 patients within 24 h of ICU admission: All patients: 3.6 (2.5–5) μmol/L Severe hypoxemia: 2.3 (2.2–2.5) μmol/L Non-severe hypoxemia: 4.3 (3.3–5.2) μmol/L [17]
		14–32 μmol/L [13]	Outpatients: 8.6 (5–14.9) μmol/L Inpatients: 2.6 (1.8–3.9) μmol/L Critically ill patients: 3.2 (2.4–4.6) μmol/L [13]
		7.8–32.3 µmol/L [19]	Mild: 6.6 (5.4–10.9) µmol/L Severe: 4.9 (4–8.1) µmol/L Critical: 5.2 (2.6–7.3) µmol/L Hospitalized survivors: 6.2 (4.3–8.0) µmol/L Hospitalized non-survivors: 4.1 (2.2–7.5) µmol/L [19]
		Reference range: 700–1800 µg/L 784.10 ± 225.60 µg/L in healthy patients [18]	Mild: 437.66 ± 294.60 µg/L Severe: 270.68 ± 116.07 µg/L Critical: 335.36 ± 206.71 µg/L [18]
			Mild: 5.0 (3.6–7.1) μmol/L Severe: 4.3 (3.1–6.2) μmol/L [21] Two months after COVID onset: Mild: 18 ± 6 μmol/L Moderate: 16 ± 6 μmol/L Severe: 15 ± 6 μmol/L [12]
Ferritin	High. High even after two months after COVID onset [12]	10–200 µg/L [17]	COVID-19 patients within 24 h of ICU admission: All patients:1476.1 (656.6–2698) µg/L Severe hypoxemia: 903.8 (566.9–2789.2) µg/L Non-severe hypoxemia: 1566.1 (729–2511.5) µg/L [17]
		30–300 µg/L [13]	Outpatients that remained home during COVID-19 disease: 227 (83–569) μg/L Inpatient cohort: 777 (341–1339) μg/L Critically ill patients: 741 (404–935) μg/L [13]
		85.2 ± 18.6 ng/mL in healthy controls [44]	Mild:135.6 ± 20.7 ng/mL Severe: 207.8 ± 45.2 ng/mL [44]
		Hospitalized COVID-19 negative patients: 231 ng/mL [135]	Hospitalized COVID-19 patients: 674 (1284) ng/mL[135]
		Reference range: 11– 336.2 µg/L Control group: 68.84 ± 43.64 µg/L [18]	Mild: 367.51 ± 196.49 µg/L Severe: 811.21 ± 847.84 µg/L Critical: 1205.77 ± 853.47 µg/L [18]
			Based on the data from 57,563 COVID-19 patients, pooled mean ferritin concentration across all ages: 777.33 ng/mL [16] COVID-19 patients at the time of hospital admission: Survivors: 503.2 (264·0–921·5) μg/L Non-survivors: 1435.3 (728·9–2000) μg/L [43] Non-severe: 708.6 ng/mL Severe: 2817.6 ng/mL [45] Mild: 363 (217–757) µg/L Severe: 1.103 (436–1.937) µg/L [21] Two months after COVID onset: Mild: 139 ± 118 μg/L Moderate: 260 ± 183 μg/L Severe: 317 ± 271 μg/L [12]
Hepcidin	High [24, 44] but also found to be lower than healthy patients in a study [18]	15.7 ± 2.6 ng/mL (healthy controls) [44]	Mild: 25.6 ± 5.8 ng/mL Severe: 31.7 ± 8.9 ng/mL [44]
		992.76 ± 230.65 pg/mL in healthy participants [18]	Mild: 894.67 ± 374.66 pg/mL Severe: 1004.69 ± 626.91 pg/mL Critical: 603.26 ± 244.37 pg/mL [18]
		Average value considered as 20 ng/mL [24]	Inpatient cohort: 91.4 (59.6–133.7) ng/mL [13]
			Two months after COVID onset: Mild: 14 ± 10 μg/L Moderate: 22 ± 14 μg/L Severe: 20 ± 13 μg/L [12]
Transferrin	Low [13, 17]. (elevated in SARS-CoV-2 infected Caco2 cells [14])	1.8–3.6 g/L [17]	COVID-19 patients within 24 h of ICU admission: All patients: 1.5 (1.1–1.8) g/L Severe hypoxemia: 1.3 (0.8–1.8) g/L Non-severe hypoxemia: 1.5 (1.1–1.8) g/L [17]
		2–3.6 g/L [13]	Outpatients that remained home during COVID-19 disease: 1.9 (1.6–2.3) g/L Inpatients: 1.5 (1.2–1.7) g/L Critically ill patients: 1.7 (1.6–1.9) g/L [13]
			Mild: 123.36 ± 23.98 ng/mL Moderate: 386.90 ± 250.33 ng/mL Severe: 478.60 ± 381.73 ng/mL [56] Mild: 2.2 g/L (mean value) Severe: 1.6 g/L (mean value) [55] Mild: 182 (155–214) mg/dL Severe: 144 (121–182) mg/dL [21] No anemia: 191 (162–230) mg/dL Anemia: 154 (129–183) mg/dL [136]
Transferrin Saturation	Low [49]	16–50% [17]	COVID-19 patients within 24 h of ICU admission: All patients: 9 (7–13)% Severe hypoxemia: 7 (6–12)% Non-severe hypoxemia: 12 (8–14)% [17]
		24.31 ± 7.18% in healthy volunteers [18]	Mild: 16.67 ± 11.67% Severe: 13.75 ± 5.94% Critical: 21.29 ± 12.99% [18]
		16–45% [13]	Outpatients that remained home during COVID-19 disease: 19 (12–28)% Inpatients: 7 (5–11)% Critically ill patients: 8 (6–10)% [13]
			In hospitalized COVID-19 patients: Mild: 14% (mean value) Severe: 16% (mean value) [55] No anemia: 11 (8–16)% Anemia: 12 (7–18)% [49] Mild: 11 (8–16)% Severe: 12 (8–19)% [21] Two months after COVID onset: Mild: 27 ± 11% Moderate: 26 ± 9% Severe: 24 ± 10% [12]
Soluble Transferrin Receptor	High in ICU patients [24]	Approximately 0.25 to 2 µg/mL in healthy controls [24]	Approximately 1.5 to 2.25 µg/mL in ICU patients [24]
		In control subjects- Within 5 days of hospital admission: 1.23 (1.05–1.41) µg/mL During 5–15 days of admission: 1.22 (1.11–1.37) µg/mL [72]	In anemic subjects: within 5 days of hospital admission: 1.13 (0.94–1.38) µg/mL during 5–15 days of admission: 1.19 (0.97–1.42) µg/mL [72]
		2–5 mg/L [70]	Two months after COVID onset: Mild: 2.9 ± 0.8 mg/L Moderate: 3.2 ± 0.9 mg/L Severe: 3.8 ± 1.3 mg/L [12]
Lipocalin-2	High	Median: 103.7 ng/mL [99]	Median values below Moderate: 118.46 ng/mL Severe: 185.40 ng/mL Critically ill: 142.830 ng/mL Deceased: 297.5 ng/mL [99]
Hemoglobin	Can be low [49, 84, 85], or at the lower end of the reference range	120–150 g/L [17]	COVID-19 patients within 24 h of ICU admission: All patients: 130.4 (20.1) g/L Severe hypoxemia: 124.7 (16.7) g/L Non-severe hypoxemia: 133.2 (21.4) g/L. All values as Mean (SD) [17]
		13–17 g/dL [13]	Outpatients that remained home during COVID-19 disease: 14 (13.2–14.9) g/dL Inpatient cohort: 13.3 (11.9–14.7) g/dL Critically ill patients: 14.3 (13.3–15.4) g/L [13]
		143.1 ± 22.2 g/L in healthy controls [44]	Mild: 136.8 ± 16.9 g/L Severe: 134.2 ± 22.1 g/L [44]
		130–175 g/L [88]	Survivors: 121 (110–129) g/L Non-survivors: 122 (106–137) g/L [88]
		138.21 ± 11.88 g/L in control group [18]	Mild: 138.37 ± 16.07 g/L Severe: 125.52 ± 16.09 g/L Critical: 98.45 ± 23.94 g/L [18]
			Based on the data from 57,563 COVID-19 patients, pooled mean hemoglobin concentration across all ages: 129.7 g/L [16] Moderate: 134.5 ± 12.1 g/L Severe:122.3 ± 27.3 g/L [89] Non-severe: 13.5 g/dL (12.0–14.8) Severe: 12.8 g/dL (11.2–14.1) [90] Non-severe: 128 (118–137) g/L Severe: 111.5 (104–128.3) g/L [91] Mild: 13.37 ± 2.19 g/dL Moderate: 13.83 ± 0.94 Severe: 11.96 ± 2.45 [56] Mild: 134 (122–145) g/L Severe: 133 (119–144) g/L [21] No anemia: 141 (134–153) g/L Anemia: 113 (101–121) g/L [136] Two months after COVID onset: Mild: 139 ± 12 g/L Moderate: 138 ± 13 g/L Severe: 139 ± 17 g/L [12]

Essentially, COVID-19 patients show lower serum iron and higher levels of serum ferritin, hepcidin, and lipocalin-2 compared to controls or the reference range. Low levels of serum iron, and high levels of serum ferritin and hepcidin were detected even after two months of COVID onset. Serum iron levels tend to decrease, while ferritin and hepcidin levels tend to increase with increasing disease severity, although the latter may not occur in all cases. Even though low serum transferrin has been observed at the time of COVID-19-related hospitalization, and the levels further decreased in those who died (associating decreasing transferrin levels with increasing severity), transferrin levels can vary during the hospital stay. Along similar lines, while TSAT has shown to decrease with increasing severity, a restoration mechanism has been observed after a few days of infection. Hemoglobin levels remain unaltered or decrease characteristically in those with hyperinflammation. Hemoglobin levels may or may not differ between the different stages of disease severity. Notably, in COVID-19 survivors, many of these iron-related parameters return to normal following discharge from the hospital (Table 1).

Generally, males are at a higher risk of severe COVID-19 than females [14]. In a study, serum iron, ferritin, and transferrin showed gender-based differences wherein the males showed greater alterations than females. It was speculated that lower serum iron and higher serum ferritin in male patients would be associated with more severe disease in male patients [13]. However, another study reported lower levels of serum iron and transferrin saturation in female patients than male patients [15]. Besides gender, age is another factor to consider in COVID-19. The risk of severe COVID-19 increases with age [14], and in COVID-19, ferritin levels increase with older age [16].

Table 2 summarizes the demonstrated and prospective usage of iron and iron-related proteins in COVID-19 prognosis.

**Table 2:** Demonstrated and prospective usage of iron and iron-related proteins in COVID-19 prognosis

Iron/iron-related proteins in serum	Proven, proposed, or potential prediction in relation to COVID-19
Iron	Hospitalization [13] Disease severity [13, 17–19] Mortality [19]
Catalytic iron	Mortality [27]
Ferritin	Hospitalization [13] ICU admission (higher frequency of ICU admission with hyperferritinemia) [50] Disease severity [18, 42, 44–46, 50] Mortality [18, 27, 43, 50, 99]
Hepcidin	Disease severity [18, 20, 44] Mortality [20]
Transferrin	Disease severity [55, 56], (transferrin: antithrombin ratio) [14]
Lipocalin-2	Mortality [99]
Hemoglobin (anemia)	Disease severity [18, 56, 85, 89, 92, 137] Mortality (anemia i.e., low hemoglobin level) [18, 49, 85]

## Iron

### Serum iron

COVID-19 outpatients and inpatients have shown low serum iron levels i.e., hypoferremia/iron deficiency (Table 1) [12, 13, 17–19]. Studies have shown that serum iron levels were below the normal range in about 90% of hospitalized COVID-19 patients [19, 20]. A study reported that 30% patients showed iron deficiency even after 60 days of the disease onset [12].

Also, iron levels showed an interesting association with oxygen demand. Although at the time of hospital admission, patients showed low serum iron, levels increased during the course of the disease in the inpatient cohort with low oxygen demand. However, in those with high oxygen demand, levels remained low, showcasing iron-related differences in patients with high and low oxygen demand [13].

### Serum iron, COVID-19 severity, and disease prognosis

A study identified serum iron level of < 6 μmol/L as a cut-off point for predicting hospitalization. Regression analysis conducted for age, gender, C-reactive protein (CRP) (inflammatory marker), and iron-related markers in the context of the requirement for hospitalization of COVID-19 patients revealed that only serum iron and ferritin were significantly associated with hospitalization. Doubling of serum iron was associated with approximately sevenfold lower odds of hospitalization [13]. Generally, low serum iron levels in the presence of infection reflect a physiological attempt to scavenge iron within the reticuloendothelial system to restrict iron availability to the growing pathogens, and thereby control the spread of infection. Therefore, in the aforementioned scenario, low serum iron is more likely to be a consequence of advanced inflammation than being a cause of hospitalization. Indeed, a factor influencing COVID-19-related hospitalization would be the patient’s pre-existing iron status and its severity, as addressed in the subsequent section.

Supporting the above-mentioned relationship between serum iron and inflammation, a study showed that compared to the outpatients, the inpatient and critically ill cohorts showed lower serum iron levels accompanied with higher CRP levels [13]. Along similar lines, severe COVID-19 showed a tendency of exhibiting lower serum iron and higher inflammation [measured via interleukin (IL)6 and/or CRP levels] than mild disease [19, 21] and this trend was observed even after 2 months of COVID-19 onset [12].

Low serum iron levels tend to show an association with increasing COVID-19 severity, but apparently only up to a certain stage of severity and not beyond. For example, low serum iron was reported in hospitalized COVID-19 patients of all disease severities (mild, severe, and critical), and those with a severe disease showed lower iron levels than those with a mild disease. Thereby, the reduction in serum iron levels was associated with progression to the severe stage (as defined in the study), and this reduction could predict disease transition from mild to severe status. However, there was no significant difference between serum iron levels of the severe and critical groups implying that there was no further reduction in serum iron levels from the severe to the critical stage [19].

Another study of hospitalized COVID-19 patients with no, mild and severe respiratory failure supported this unusual pattern. Serum iron levels in patients with mild respiratory failure were significantly lower than those without respiratory failure. However, there were no significant differences in levels between patients with severe respiratory failure and no respiratory failure. In other words, from the patient groups with no, mild, and severe respiratory failure, lowest serum iron status was found in COVID-19 patients with mild respiratory failure, while patients with no respiratory failure and severe respiratory failure showed comparatively higher serum iron levels. This demonstrated a U-shaped relationship between serum iron levels and disease severity [15]. The drop in serum iron levels as the severity progressed from no respiratory failure to mild respiratory failure could be related to the natural innate iron-lowering response to infection/inflammation. However, higher iron levels in those with severe respiratory failure compared to those with mild respiratory failure indicates that the increment in iron may play a role in the pathological progression of the disease. This could partly explain the reason for higher serum iron levels (not low serum iron levels) to be associated with the development of severe respiratory failure [15].

Furthermore, no significant difference in serum iron levels was observed between hospitalized survivors and non-survivors [18–20]. It is possible that while differences in serum iron levels (reflected as the aforementioned U-shaped association between iron and disease severity) can be observed during the early stages of the disease, those differences diminish as the disease progresses to the critical stage and involve an increment and restoration of iron levels, resulting in similarities in levels between survivors and non-survivors. Whether this restoration contributes to aggravation of disease pathology remains to be clearly determined.

It is important to consider that generally the levels of serum iron vary remarkably between individuals and can vary every hour. Even when measured in combination with transferrin iron-binding capacity, it is not greatly reliable because of its fluctuations and alterations induced by infection and any recent intake of iron [22]. Low iron levels have been found in iron deficiency as well as post-surgery anemia of chronic disease [23]. Thus, using serum iron for clinical evaluation of iron status is cofounded by several limitations and must be implemented with caution.

### Catalytic iron contributes to COVID-19 pathogenesis

Non-transferrin-bound iron or non-ferritin-bound iron is generally referred to as catalytic iron or free iron. In COVID-19 patients, free iron levels were elevated along with reduced levels of glutathione, the antioxidant [24]. Free iron can accelerate the Fenton reaction to produce increased levels of reactive oxygen species that can eventually damage cells/tissues. Catalytic iron is associated with oxidative stress, vascular injury [25] and increased risk of mortality in patients with acute kidney injury [26]; the latter is one of the complications in COVID-19. This free-iron-induced oxidative stress may contribute to the multi-organ dysfunction in COVID-19 and exacerbate pathology. Thus, in hospitalized COVID-19 patients, catalytic iron levels have been positively associated with in-hospital mortality and adverse clinical outcomes [27]. Furthermore, it has been speculated that SARS-CoV-2 may require iron for replication and other functions, although this proposition has been challenged by some. If true, then the availability of free iron might induce greater pathogenicity [27, 28].

### Iron may help sustain mucormycosis in COVID-19

Glucose-regulated protein-78 (GRP78) is an endoplasmic reticulum chaperone protein that is overexpressed on the cell membrane during stressful conditions, viral infections, hyperglycemia, and hyperferritinemia. It is an important mediator of SARS-CoV-2 entry [29] and a facilitator for infection by *Mucorales* (fungal group) causing mucormycosis [30]. Diabetes is highly prevalent in India, and the association of overexpression of GRP78 with hyperglycemia may partly explain the reason for the twofold increase in COVID-19-associated mucormycosis in India after COVID-19 onset [31]. However, this needs further investigation, particularly because the estimated prevalence of mucormycosis in India was higher than that in other countries even before the pandemic. This may have been due to the high numbers of diabetics, unbiased usage of corticosteroids and health-care issues [32, 33].

Mucormycosis is mostly associated with diabetic ketoacidosis [30]. SARS-CoV-2 infection may cause ketosis and ketoacidosis [34]. Acidosis releases iron from iron-binding proteins [30]. At low pH (below 7.4), the ability of transferrin to bind to iron is greatly reduced [35]. This results in increased amount of non-transferrin-bound iron in the blood during acidosis. The availability of iron improves the growth and survival of *Mucorales* in the human host [30]. Since acidosis also elevates GRP78 in nasal sinuses and lungs [30], diabetic ketoacidosis is one of the risk factors for mucormycosis in COVID-19 patients [36].

Iron-rich nanoparticles (15–40 nm diameter) have been found in polluted air [37]. Air-pollution-induced endoplasmic reticulum stress can upregulate GRP78, leading to increased susceptibility to both SARS-CoV-2 infection and mucormycosis.

Thus, both, iron release by acidosis and iron-rich polluted air can elevate the risk of mucormycosis in COVID-19 patients.

## Ferritin

Ferritin is an iron-storage protein found in the circulation, cytosol and within the mitochondria. While ferritin synthesis increases with increased cellular iron levels [38], serum levels of ferritin increase in response to systemic inflammation, in addition to the response to elevated iron levels. Thus, serum ferritin has been extensively used as a marker to assess iron status, infection or inflammation, malignancy [39] and autoimmune conditions [40].

Notably, ample hepatic ferritin deposition was observed in severe COVID-19 patients, which is likely responsible for liver injury in these patients [41]. In COVID-19 patients, serum ferritin was greatly increased (hyperferritinemia) compared to COVID-19 negative controls (Table 1) [42]. Elevated serum ferritin in concomitance with hypoferremia indicates that the elevation in ferritin is a response to COVID-19-induced hyperinflammation and not iron loading. Notably, in COVID-19 patients discharged from the hospital, ferritin levels were found to decrease/normalize to 142 μg/L (64–269 μg/L) in around 122 days (median) after discharge [21].

### Ferritin, COVID-19 severity, and disease prognosis

Ferritin levels demonstrate a positive correlation with disease severity, i.e., a trend of increasing ferritin levels with increasing severity [12, 18, 43–47]. Persistent hyperferritinemia was more common in severe/critical COVID-19 than mild disease [12]. Serum ferritin levels were higher in critical patients (compared to those with mild/moderate severity), severe patients (compared to non-severe patients), non-survivors (compared to survivors), inpatients and critically ill patients (compared to outpatients), and in those patients requiring ICU and mechanical ventilation (compared to those who didn’t require these) [13, 42–45]. Thus, ferritin could be used to distinguish between severity stages. Also, levels were higher in intubated and deceased patients [18] and in those with respiratory failure (compared to those without) [15]. Essentially, ferritin levels significantly associated with hospitalization [13], need for mechanical ventilation, kidney replacement therapy, and thereby with adverse clinical outcomes in COVID-19 [27].

Ferritin is not only a marker of disease severity [27, 42] but also one of the independent predictors of disease severity [48]. For example, COVID-19 severity could be predicted when serum ferritin levels surpassed 162 ng/mL (86.9% sensitivity, 70.3% specificity) [44]. A ferritin/transferrin ratio greater than 10 predicted a 5-times higher risk of admission in ICU and 8-times higher risk of requiring mechanical ventilation [49]. However, in one study, serum ferritin levels were very variable and could not differentiate between patients requiring high and low oxygen [13].

Increased ferritin levels were associated with increased COVID-19-related mortality [12, 27], and thus high serum ferritin was an independent predictor of in-hospital COVID-19-related mortality [50]. Non-survivors showed higher mean ferritin levels than COVID-19 survivors. Many patients with raised serum levels of ferritin (> 300 μg/L) had a much higher likelihood of death before discharge [43].

## Hepcidin

The iron-hormone hepcidin regulates systemic iron homeostasis in the body. It is a 25-amino acid peptide that is secreted into the circulation in response to inflammation and systemic iron elevation. Hepatocytes are the predominant source of circulating hepcidin [51].

COVID-19 patients showed elevated serum hepcidin levels [13, 24, 44]. In a study, hepcidin levels were elevated in about 60% of hospitalized COVID-19 patients [20].

### Hepcidin, COVID-19 severity, and disease prognosis

High hepcidin levels positively associated with severe COVID-19. Hepcidin measured at the time of hospitalization predicted the clinical outcome [20]. Patients with severe disease showed higher hepcidin levels than those with mild disease, and significant differences in levels were observed between mild, severe, and healthy patients [44]. Thus, hepcidin levels could be used to distinguish between the severity stages of COVID-19.

Hepcidin level predicted disease severity [20, 44]. It predicted the development of severe pneumonia in COVID-19 where the level of 32.7 ng/mL defined the ‘highest limit of cases without severe pneumonia’. Combined detection of serum ferritin and hepcidin even better predicted (showed best sensitivity and specificity) COVID-19 disease severity [44]. In hospitalized COVID-19 patients, baseline levels of serum hepcidin were associated with the need for mechanical ventilation and kidney replacement therapy, and thus adverse outcomes. Also, serum hepcidin levels were associated with in-hospital mortality. Non-survivors showed higher baseline levels of hepcidin [27]. COVID-19 patients with hepcidin levels lower than 394 ng/mL had the lowest probability of mortality. In critical patients admitted to ICU, high hepcidin levels predicted mortality [20].

Distinct from the above, a study showed that critically ill COVID-19 patients in ICU had lower serum hepcidin levels than healthy patients. Levels were much lower in the intubated group than in non-intubated group. In patients with mild and severe disease, levels did not majorly deviate from those in healthy patients and no difference in levels were observed between survivors and non-survivors [18]. In another study, hepcidin levels did not show a correlation with serum iron levels, contrasting the norm. Also, hepcidin levels could not distinguish between patients with high and low oxygen demand in hospitalized patients [13].

## Transferrin

Transferrin is a glycoprotein that is synthesized mainly in the liver and secreted into the circulation. Here, it functions as an iron-carrier protein. It binds to transferrin receptors on cell surfaces and delivers transferrin-bound iron to the cells via receptor-mediated endocytosis [52]. Normally, transferrin is upregulated by iron deficiency [53] and hypoxia [54], probably as a mechanism to increase iron availability to the developing erythrocytes, and it is downregulated by inflammation [53], likely to reduce iron availability to the growing pathogens.

Serum transferrin levels were low in COVID-19 outpatients and inpatients (Table 1) [13, 17, 55]. This occurred despite low levels of serum iron, which implied that COVID-19-induced inflammation exerted dominance over transferrin-inducing mechanisms and prevented its elevation under low iron conditions. However, another set of data indicated that transferrin expression was upregulated in SARS-CoV-2 infected Caco2 cells [14]. The reason for this contrast needs to be clarified.

### Transferrin and COVID-19 severity

In a COVID-19-related study, transferrin levels were low at the time of hospitalization and a decrease in transferrin levels persisted more strongly in patients with high oxygen demand over the time of hospitalization (examined up to 6 days) [13]. Along similar lines, in hospitalized COVID-19 patients, within the first week of hospitalization, transferrin levels were found to decrease in all patients. The decrement continued in those patients who died. Thus, low levels of transferrin can pose a risk for mortality in hospitalized COVID-19 patients. Notably, in COVID-19 patients discharged from the hospital, transferrin levels were found to increase/normalize to 253 mg/dL (231–283 mg/dL) in around 122 days (median) after the discharge [21].

Compared to the outpatients, the inpatients and critically ill patients showed lower transferrin levels [13]. COVID-19 patients with severe disease showed significantly lower mean transferrin values than those with mild disease. Here, low transferrin levels predicted increased inflammation and disease severity [55]. These data presented a negative association between serum transferrin levels and disease severity. However, in a different study, transferrin was significantly lower in mild cases compared to moderate and severe cases, and its levels positively were correlated with computed tomography scores that were indicative of COVID-19 severity [56]. In another study involving COVID-19 patients in ICU, transferrin levels showed non-significant subtle alterations (both increment and decrement) through the period of 18 days in ICU, with subtly higher levels on days 15–18 compared to days 1–2 of the ICU stay. While the levels on days 15–18 were within the normal range, alterations on previous days spanned the lower end of the spectrum of the normal range [57].

In addition to functioning as an iron-carrier in the circulation, transferrin is a procoagulant. It interferes with the antithrombin/SERPINC1-facilitated inhibition of coagulation proteases such as thrombin and factor XIIa, and thereby increases coagulation [58]. Thus, transferrin may play a role in coagulation in patients with severe COVID-19 because the severe form of this disease has been linked with disseminated intravascular coagulation and thrombosis [14]. Also, locally produced transferrin may aggravate COVID-19 pathology. For instance, high levels of transferrin produced by the brain may lead to hypercoagulation and ischemic stroke. Hemorrhagic and ischemic strokes are some common COVID-19-related complications [14].

Analyses of data from Genotype-Tissue Expression database showed that transferrin levels were higher in males than in females and the levels increased with age, unlike antithrombin that did not differ between males and females and did not increase with age. Therefore, transferrin/antithrombin ratio showed increment with age and was higher in males, which corelated with the risk for severe COVID-19, the risk being higher in males and increases with age. Thus, it was proposed that transferrin/antithrombin ratio may play an important role in COVID-induced coagulopathy in older males with severe disease [14].

## Transferrin saturation

Transferrin saturation with iron (TSAT) indicates the amount of iron bound to transferrin. Transferrin has two iron-binding sites and its occupancy with iron ranges between 20–45% [51]. Thus, TSAT is a crucial marker of systemic iron status, and it reflects iron bioavailability.

Compared to the normal range and healthy volunteers, COVID-19 patients showed lower TSAT [13, 17, 18, 55].

### TSAT and COVID-19 severity

Outpatients that stayed home during the course of the disease showed TSAT towards the lower end of the normal range. TSAT was characteristically below the normal range in the inpatients and the critically ill, and lower in these groups compared to the outpatients (Table 1) [13]. In a study, TSAT was highly reduced at ICU admission (median 9%), and then it increased between the 3^rd^ and 6^th^ day of admission (median 33%). This showed restoration of TSAT levels. A pattern of low TSAT in the early stage of infection followed by an increment in the later stage to return to normal within 7–10 days is generally observed during infections whereby the aim is to limit the bioavailability of iron for the growing pathogen in the early stages [57]. This is supported by several studies. For example, although the ICU patients showed lower TSAT compared to healthy volunteers, it was lower in the mild and severe patient groups but not in the critically ill patients. In the latter group, mean TSAT value was almost the same as that in the healthy volunteers. This shows a TSAT restoration mechanism after some time of infection [18]. Similarly, TSAT was markedly higher in patients with severe respiratory failure than in patients with mild or no respiratory failure, levels being markedly lower in female patients relative to male patients [15]. Intubated patients had higher TSAT than non-intubated patients [18], which could be due to differences in iron regulation at different stages of disease severity or an effect of intubation on iron parameters.

Generally, an increase in TSAT over time is likely due to the decline of transferrin levels, particularly in ICU/critically ill patients [59, 60]. Such a decline in transferrin levels has been observed in severe COVID-19 compared with mild disease [55]. In ICU patients with sepsis, high TSAT has been associated with poor survival [59]. This could be related to the fact that increased TSAT enhances iron availability to the pathogens. Thus, ICU patients are more likely to die from sepsis with high TSAT but low transferrin at the time of admission [59, 60]. Bearing that TSAT tends to decrease during inflammation [60], the observed decrements and increments in TSAT during the course of COVID-19 appear to be predominantly driven by inflammation. This is not surprising because its components (iron and transferrin) are regulated by inflammation.

While TSAT is generally a good predictor of outcome for ICU patients [60], its utility in evaluating COVID-19 needs to be assessed. This is partly because of its variability over time and some other observations such as no significant differences in TSAT i) between non-severe hypoxemic and severe hypoxemic COVID-19 patients within 24 h of admission to ICU [17], ii) between anemic and non-anemic COVID-19 patients [49] and iii) between mild, moderate and severe disease after two months of COVID-19 onset. A proportion of patients (20%) did show combined anemia of inflammation and iron deficiency anemia. The latter was defined as low TSAT (< 20%) together with low ferritin (< 30 µg/L), among other parameters [12].

## Membrane-bound transferrin receptor

### Contextual background: SARS-CoV-2 entry into host cells

Structural data has revealed that the viral spike protein has a receptor-binding domain. The residues in the receptor-binding region have high affinity for the angiotensin-converting enzyme-2 (ACE2) receptors present on the type II alveolar epithelial cells of lungs (airway way and the alveolar cells). Through its spike proteins, SARS-CoV-2 attaches to the host’s ACE2 receptor and enters the host cell. Essentially, following the binding of the receptor-binding domain to the ACE2 receptors, SARS-CoV-2 is endocytosed in the epithelial cell, released, and undergoes rapid replication. This leads to a virus-associated programmed cell death. Viral RNA is released, and the damage and death of epithelial cells trigger an inflammatory response in the lungs that cause acute respiratory distress syndrome and fibrosis [61–64]. Diabetes, cardiovascular disease, and hypertension are risk factors for COVID-19. These patients have upregulated ACE2 receptors, which facilitates viral entry and promotes infection in this group. Also, normally, the ACE2 receptors help in regulating inflammation. Binding of the viral proteins to the ACE2 receptors inhibits this functionality of these receptors [2], which can further aggravate the disease. Notably, silent hypoxia is prevalent among COVID-19 patients and hypoxia can increase ACE2 expression in the early stages of the disease and thereby damage lung cells. However, its expression decreases to baseline levels during the later stages, which can help in disease amelioration [65].

### Putative role of transferrin receptor in viral entry

Transferrin receptor-1 (TfR1) is ubiquitously expressed in several tissues and cell types, including cells of the respiratory tract. It binds to transferrin (iron-bound) in the circulation and through endocytosis of the complex, transferrin-bound iron uptake is mediated. Through this mechanism, iron is acquired by various cell types for their activities [52].

Interestingly, SARS-CoV-2 has shown to infect ACE2-negative cells, indicating the existence of other routes of viral entry [66]. It was previously suggested that several viruses recognize and bind to TfR1 on its apical domain and target the endosomal compartment. TfR1 characteristics such as its ubiquitous nature and its ability to be recycled back to the cell surface (for further iron uptake) support viral infection [67]. Since TfR1 can allow entry of viruses into host cells, SARS-CoV-2 entry via TfR1 is indeed possible.

In vitro studies have shown evidence of TfR1 acting as an alternative receptor for SARS-CoV-2 entry. Direct interaction of TfR1 and SARS-CoV-2 was shown by producing docking models of TfR-ACE2 and TfR-ACE2-spike protein interactions. Also, in SARS-CoV-2 infected cells, TfR1, ACE2 and SARS-CoV-2 colocalization was found on the cell membranes, and TfR1 and SARS-CoV-2 colocalization was seen in the cytoplasm, indicating that TfR1 may be involved in transporting the SARS-CoV-2 virus into the cytoplasm [68]. Therefore, TfR1 is seen as a potential therapeutic target to block viral entry and experiments are being conducted to assess this approach.

Ferristatin II selectively induces TfR1 internalization and degradation. In a study, it significantly inhibited SARS-CoV-2 replication in/infection of Vero cells. It was hypothesized that ferristatin II blocks TfR1-mediated SARS-CoV-2 entry into the host cells [69]. The effect of ferristatin II on ACE2 receptors, the known receptors of SARS-CoV-2, is yet to be determined. However, based on the observations, the authors proposed that some SARS-CoV-2 receptors on the host cells may remain functional and bind to virus, but since TfR1 was absent on the cell surface (due to ferristatin II- induced degradation), the virus (i.e., receptor-binding domain of the viral spike protein) was not incorporated in the host cells [69]. Therefore, inhibiting the virus-TfR1 interaction could reduce SARS-CoV-2 infection.

## Soluble transferrin receptor

Soluble transferrin receptor (sTfR) is a truncated and cleaved form of membrane-bound TfR-1. It is found in the circulation. The main source of circulating sTfR is the erythroid repertoire [70]. Iron status alters serum sTfR levels. These are increased in iron deficiency anemia but stay within the normal range in anemia of inflammation associated with chronic diseases. This helps in differentiating iron deficiency anemia from anemia of inflammation [71].

Compared to healthy volunteers, sTfR concentrations were higher in COVID-19 patients admitted to ICU [24]. This matches with the characteristically low serum iron in severe COVID-19 (Table 1) and indicates that the increment in sTfR may be the result of serum iron deficiency in these patients. Interestingly, another study showed no significant differences in levels of sTfR in COVID-19 patients in ICU for up to 15-18 days [57]. Also, there were no significant differences in levels between control and anemic patients at two time points: within 5 days and during 5-15 days of hospital admission (Table 1) [72]. The reason for such differences in sTfR levels needs to be investigated. Two months after COVID onset, sTfR levels showed significant differences between mild, moderate and severe cases, where levels in the severe group were higher than in the other two groups (Table 1) [12].

## Lactoferrin

Lactoferrin is a glycoprotein that belongs to transferrin family, and like transferrin, it is an iron-binding protein. It is found in most body fluids and shows antimicrobial (antibacterial, antiviral, and antifungal), anti-inflammatory, and immune-regulating properties [6, 73].

Lactoferrin has shown the potential to tackle SARS-CoV-2 infection [74]. It is a broad-spectrum antiviral agent [74], and it is effective against many viruses [75–77]. Based on previous studies, Lang et al. discussed that lactoferrin can promote the aggregation and adhesion of neutrophils against SARS-CoV [76]. Also, it can enhance natural killer cell activity through the production of IL-18 and Type 1 interferons (in mice) [78]. Interestingly, in an in vitro study prior to the COVID-19 pandemic, lactoferrin inhibited the entry of SARS-CoV pseudovirus in HEK293E/ACE2-Myc cells in a dose-dependent manner. It did not impede the interaction of the spike protein with the ACE2 receptors but inhibited the binding of the spike protein to HEK293E/ACE2-Myc cells by binding to the heparan sulfate proteoglycans (HSPGs) on the host cell membranes [76].

Lactoferrin has been proposed to inhibit the virus or viral entry via several mechanisms. First, lactoferrin is a highly positively charged protein. This promotes its binding to negatively charged surfaces of viruses and other microorganisms. Thus, it may directly bind to the virus and prevent its entry. Second, binding of lactoferrin to its receptor on the cell surface induces intracellular signals leading to increased interferon synthesis which blocks viral replication. Third, the virus attempts to enter the host cell by initially binding to HSPGs that are located on surfaces of cell membranes of the host cells. In doing so, the virus increases its likelihood of binding to a more specific receptor thereafter. Binding of lactoferrin to the HSPGs prevents the binding of the virus to the HSPGs and thus, viral entry is prevented. In addition, since lactoferrin has immunomodulatory/anti-inflammatory properties, it is believed to help in tackling the cytokine storm in COVID-19 [74, 79, 80]. Thus, based on these data, lactoferrin treatment as an adjunct treatment to tackle SARS-CoV-2 infection can be envisaged. Clinical trials need to be conducted to affirm this potential of lactoferrin.

## Hemoglobin function and level

Human adults have a total body iron content of around 3–4 g. Approximately 70% of the body iron (about 2–3 g) is bound to hemoglobin in red blood cells (RBCs), which makes these cells the largest ‘consumers’ or ‘holders’ of iron in the body. Hepatocyte iron stores represent the second largest iron compartment which contains about 1 g of iron [51].

### Proposed alteration in hemoglobin function

Pathophysiological mechanisms have been proposed regarding the interaction of the virus with hemoglobin. According to the proposition, SARS-CoV-2 first binds to ACE2, CD26, CD147 and other receptors on the surfaces of RBCs (and/or blood cell precursors). Due to the interaction between viral spike proteins and cell-surface receptors, the virus is endocytosed. Viral ORF8 protein and surface glycoprotein mediate viral interaction with hemoglobin. The virus binds to porphyrin and attacks heme on hemoglobin’s beta-1 chain. Subsequently, hemolysis is induced with eventual dissociation of iron from the complex. Also, the virus may bind to the released heme to form a complex, which may generate dysfunctional hemoglobin with reduced oxygen and carbon dioxide binding abilities. Inhibition of heme metabolism due to the binding of viral ORF8 protein to porphyrin has been suggested [81–83]. These events would adversely affect oxygen delivery to crucial organs and accelerate multi-organ failure. Hereby, SARS-CoV-2 infection is believed to alter hemoglobin function.

### Normal or reduced hemoglobin levels in some patients

COVID-19 patients have shown hemoglobin levels that were normal [19] or low or at the lower end of the reference range (Table 1).

In hospitalized patients, decreased hemoglobin levels were reported in about 20% patients (< 110 g/L) [48] or about 50% patients [72, 84, 85]. Low hemoglobin level (< 12.5 g/dL) was more common in non-surviving patients than surviving patients in Italy’s first COVID-19 wave [86]. A study showed that in hospitalized COVID-19 patients without hyperinflammation, hemoglobin levels remained almost unaltered. However, in patients with hyperinflammation, hemoglobin levels decreased rapidly, and the decrement was highly evident in those patients that showed hyperinflammation upon hospital admission. Hyperinflammation was associated with an elevated risk for new-onset anemia within a week of hospital stay, and new-onset anemia during hospitalization posed a risk for ICU admission [21]. Anemia in COVID-19 patients (as characterized by reduced hemoglobin levels) has been independently associated with disease severity and poor outcomes including ventilator requirement, ICU admission and high in-hospital mortality [16, 49, 85].

### Hemoglobin and COVID-19 severity

In the context of severity-based differences in hemoglobin levels, some studies showed no major differences in levels between mild, moderate, and severe cases [12], outpatients, inpatients, and critically ill patients [13], healthy controls and patients with mild and severe COVID-19 [44], patients with non-severe and severe hypoxemia [17], non-ICU and ICU patients at the time of admission [87], and survivors and non-survivors [88] (Table 1).

In contrast, in other studies, patients with severe disease showed a distinct drop in hemoglobin levels [21], severe cases showed significantly lower hemoglobin levels than mild and moderate cases [56], moderate cases [89], and non-severe COVID-19 patients (Table 1) [90, 91]. Apparently, hemoglobin levels decreased with disease severity; being lower in critically ill and deceased patients, and in ICU patients compared to non-ICU patients (nadir) [18, 87]. Essentially, levels were lower in critical and deceased patients [18, 92, 93], in older patients and in those with cardiovascular disease, hypertension and diabetes [16], the common comorbidities found in COVID-19 patients aged between 70 and 80 years [88]. While hemoglobin levels in the COVID-19 inpatients were lower than outpatients, levels were found to be consistently lower in patients with high oxygen demand [13].

Hemoglobin-associated markers could assess COVID-19 severity and aid in diagnosis and prognosis. For example, significantly increased levels of bilirubin were detected in ICU patients compared to non-ICU patients [94]. Among other parameters, higher level of direct bilirubin was associated with severe disease {non-severe Median (IQR): 3.9 (2.7–5.2) µmol/L, severe Median (IQR): 5.2 (3.4–7.8) µmol/L} and could predict disease severity [95]. Hemoglobin, RBC count and hematocrit were significantly lower in the severe group than in patients with moderate severity [89]. Thus, it was possible to distinguish between moderate and severe cases based on hemoglobin levels and other blood parameters.

In hospitalized COVID-19 patients with mild and severe disease, hemoglobin levels declined over the two weeks of hospitalization but then normalized and increased to 136 g/L (118–146 g/L) and 146 g/L (128–151 g/L), respectively, in around 122 days (median), after being discharged [21].

## Lipocalin-2

The lipocalin family consists of small proteins which regulate the immune system and act as transporters of various substances such as retinoids, fatty acids, and steroids. Lipocalin-2 is a part of this family. It is secreted by various cells and tissues under pathological and physiological conditions, and it possesses antifungal and antibacterial properties. Lipocalin-2 is an iron-binding cytokine. Its ability to bind to iron reduces iron availability to the pathogens, thereby hindering their growth. Lipocalin-2 also protects against oxidative stress induced by excess free iron [96, 97].

Serum lipocalin-2 levels were higher in symptomatic and asymptomatic COVID-19 patients compared to healthy volunteers/control group [98, 99]. Levels were raised in severe and critical cases (compared to healthy controls), but no remarkable differences in levels were observed between patients of varying severities, and no significant change in levels were seen in critically ill patients in ICU compared to patients recovering from COVID-19 post the ICU stay [99]. Also, levels were not further elevated in COVID-19 patients that required ICU admission compared to those who didn’t, so serum lipocalin-2 was not found to be an efficient predictor of ICU admission of COVID-19 patients [100]. Compared to healthy controls, levels were higher in deceased patients and elevated levels of serum lipocalin-2 were associated with mortality [99].

## Iron chelation as a therapy for COVID-19: A highly debatable topic

Several authors have suggested the usage of iron chelators to ameliorate COVID-19 symptoms [101]. Abobaker proposed that in COVID-19 patients, iron chelation could improve clinical outcomes. This suggestion was based on the idea that binding of SARS-CoV-2 to hemoglobin would release iron from heme, and this would increase free iron in the body. Reactive oxygen species would be generated, and these would contribute to pulmonary damage. Therefore, this excess free iron, which can cause oxidative stress, needs to be scavenged via iron chelation. The author suggested that the increment is serum ferritin in COVID-19 patients was due to increase in heme-derived free iron. Also, iron overload increased viral replication and therefore chelating iron might help in reducing disease severity [102]. These opinions were likely informed by the data that pulmonary iron loading accelerates the pathogenesis of pulmonary fibrosis, contributes to lung function decline, and this can be reduced by using the iron chelator deferoxamine, as shown in mice models [9].

However, others suggested that iron chelation may not benefit COVID-19 patients. Several reasons for this were put forward. First, during SARS-CoV-2 infection (and any other infection), the body’s natural innate immune response is to sequester iron to induce temporary iron deficiency so that iron is not available to the replicating virus/microorganism and the infection could be controlled. In this scenario, the usage of iron chelators may further reduce iron levels in the body and may be injurious if the patients suffer from anemia of inflammation.

Secondly, it was suggested that SARS-CoV-2 is an RNA virus and does not use iron. Instead, it uses RNA duplex intermediate for replication. So, chelating iron with the expectation that this will decelerate viral growth/replication may not work. Thirdly, the idea that SARS-CoV-2 attacks the hemoglobin chain leading to a decrement in hemoglobin levels needs more evidence because the binding of the virus to hemoglobin was predicted and this prediction was based on docking models/bioinformatic analyses. Low hemoglobin was observed only in some patients, and in some cases, the decrement was subtle.

Therefore, it was suggested that iron chelation should not be applied unless elevated iron levels are demonstrated in patients and these are caused by SARS-CoV-2 infection [103].

Hypoxia increases pulmonary hypertension [104]. Hypoxic pulmonary vasoconstriction (HPV) is an inherent homeostatic mechanism of the pulmonary circulation that involves constriction of pulmonary arteries of hypoxic lung sections in response to alveolar hypoxia. The aim of this mechanism is to divert blood to better-ventilated alveoli to optimize ventilation/perfusion matching and maintain oxygenation [105].

In COVID-19, HPV is impaired. Impaired HPV together with high-altitude pulmonary edema and ARDS cause hypoxemia in COVID-19 patients [105]. In COVID-19-related ARDS, there is major arterial oxygenation impairment, implying an inappropriate pulmonary vascular response to hypoxia [15].

Pulmonary arterial pressure and pulmonary vascular response to hypoxia is influenced/altered by iron. In healthy volunteers, iron loading under hypoxic conditions reduced or abolished the sensitivity of pulmonary vasculature to hypoxia, whereas iron chelation increased pulmonary artery systolic pressure and pulmonary vascular response to hypoxia [104]. It is postulated that the increment in iron levels during the advanced stages of COVID-19 is associated with impaired HPV and the subsequent hypoxemia. Therefore, it has been suggested that in COVID-19 patients with severe respiratory failure, reducing serum iron levels could augment arterial oxygenation [15] and thereby reduce hypoxemia.

While these data leave us in ambiguity on whether iron therapy or iron chelation could be useful, the data affirm the significance of iron in COVID-19 pathology. Indeed, if iron-related therapy (chelation or administration) were to be used, then further investigation would be required to determine optimal levels of iron and chelators for the treatment.

## Interplay of iron parameters in COVID-19

Reported iron-associated observations in COVID-19 include anemia of inflammation, decrements in serum iron (i.e., hypoferremia), transferrin and transferrin saturation (TSAT), and increments in serum ferritin (i.e., hyperferritinemia), hepcidin, lipocalin-2, catalytic iron, and soluble transferrin receptor. Hemoglobin levels can be low or normal, and hemoglobin function is believed to be compromised.

**Fig. 1:**
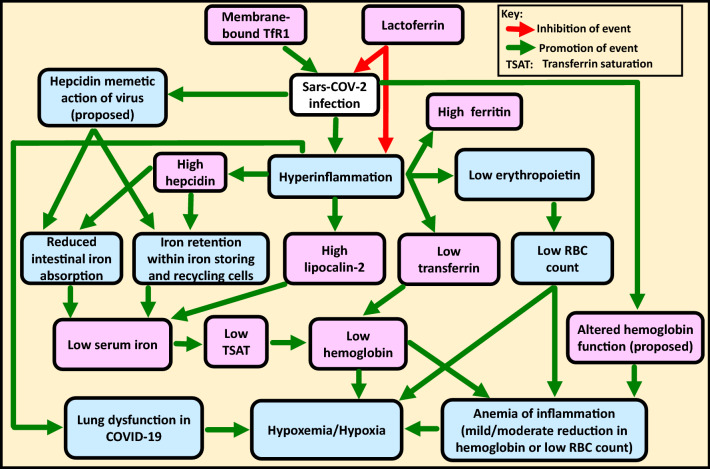
**Interplay of iron-related parameters in COVID-19**. The figure shows the associations and effects of iron-related parameters on each other in COVID-19 and proposes how these could contribute to disease pathology

In COVID-19, hyperinflammation caused by the cytokine storm appears to be the main driver of pathology and the observed iron-related alterations (including anemia) [12]. Figure 1 summarizes the associations and effects of these iron-related parameters on each other in COVID-19 and depicts how these could possibly contribute to COVID-19 pathology. When the infection is cured/controlled, normal iron homeostasis is reestablished [21].

### What elevates serum hepcidin and ferritin in COVID-19? Inflammation

COVID-19 patients show hyperinflammation. Levels of interleukins (IL) 2,7,10, MCP1, TNFα, MIP1-α, GSCF and IP10 are higher in ICU patients than in non-ICU patients [106]. These patients, particularly those with severe and critical disease (those with high oxygen demand) show high levels of IL6, which appears to play the most important role in pathology. Its increased level in serum corelates with ARDS, respiratory failure and worse clinical outcomes [13, 107].

Hepcidin is induced by infection/inflammation, specifically by IL6 via the JAK/STAT signaling pathway [108]. Other cytokines like IL1, IL22, and interferon-α also potentiate hepcidin induction [109]. Thus, in COVID-19, hepcidin level is elevated (Table 1), and this elevation is due to COVID-19-induced inflammation. High serum hepcidin level, particularly at the time of hospital admission likely reflects the high level of inflammation and hyperferrtinemia. IL6, hepcidin and ferritin levels were positively correlated in a group of COVID-19 patients [20, 21].

Likewise, in addition to elevation by iron [38], serum ferritin increases in response to inflammation [110], as observed in COVID-19. Thus, the increments in serum hepcidin and ferritin in COVID-19 are attributed to heightened inflammation.

### What is the result of elevated hepcidin? Hypoferremia

High levels of circulatory hepcidin inhibit intestinal iron absorption and lock iron within the iron-storing and iron-recycling cells by degrading the cellular-iron transporter ferroportin. This prevents iron entry and subsequent iron elevation in the circulation [111]. Thus, inflammation-induced hepcidin increment leads to systemic hypoferremia (low serum iron levels) [112]. In addition, structural similarity has been identified between hepcidin and SARS‐CoV‐2 spike protein. It has been hypothesized that the viral spike protein may have a hepcidin-mimetic action and induce ferroportin blockage [81]. If correct, then this can further promote intracellular iron retention and contribute to systemic hypoferremia.

This iron-lowering iron-sequestering response is not a COVID-19-specific response but a generic response to infection. In the early stages of infection, the aim is to sequester iron and reduce its availability to the growing pathogens. Serum iron and TSAT decrease in the early phase of infection but return to normal in about 7–10 days [57]. This reflects the body’s innate immune response (defense mechanism) against microbial invasion [112].

Interestingly, low serum iron levels are associated with elevated thromboembolic risk in patients with hereditary hemorrhagic telangiectasia [113]. COVID-19 patients are at high risk of thromboembolism [114]. Thus, low serum iron in COVID-19 patients may contribute to this risk.

### Low serum hepcidin in COVID-19. What may be the cause?

Contrasting the studies that showed elevated hepcidin in COVID-19, a study showed low hepcidin levels in critically ill COVID-19 patients [18]. This is interesting because hepcidin is elevated by inflammation and COVID-19 is characterized by hyperinflammation. Moreover, presence of low levels of the hepcidin suppressor erythropoietin in critically ill COVID-19 patients (compared to healthy patients [18] should allow hepcidin induction. So, reduced hepcidin level is an unexpected response in this situation. This implies that in these critically ill patients, probably, the effect of low erythropoietin on hepcidin synthesis was minor/secondary and other hepcidin-regulating factors took precedence, resulting in hepcidin downregulation. Bearing that hepcidin elevation in COVID-19 has been observed in several studies (Table 1), it is possible that the hepcidin response (elevation or downregulation) in critically ill COVID-19 patients is a result of a tug-of-war between hepcidin inducers and suppressors and may also depend on the stage of the disease at which the measurements are taken.

Generally, hepcidin is suppressed by hypoxia [115]. Silent hypoxia is prevalent among COVID-19 patients [65], and this suggests the possibility that hepcidin suppression could be partly due to hypoxia, at least at a certain stage of infection and among certain COVID-19 patients. However, under the strong influence of virus-induced inflammation, which increases hepcidin levels (Table 1), hepcidin suppression seems less likely. But, hypoxia can reduce inflammation-induced hepcidin synthesis [116]. Thus, it is possible that while the inflammation may induce hepcidin synthesis, hypoxia may attenuate it, leading to the observed decrease in hepcidin levels in this study.

It is possible that anemic COVID-19 patients may be suffering from a pre-existing illness that cause blood loss in these patients via gastrointestinal or urinogenital bleeding. Also, iatrogenic blood draws lead to loss of blood. In a study, COVID-19 patients in ICU showed a loss of about one third of the total patients’ red blood cell mass because of repeated blood sampling during the ICU stay [117]. Moreover, these patients may be suffering from pre-existing iron deficiency. These factors can reduce hepcidin levels and could be the reason for low hepcidin levels in anemic COVID-19 patients [118–120].

### What are the plausible causes of hypoferremia in COVID-19 in the absence of hepcidin elevation?

Hypoferremia in COVID-19 is primarily attributed to elevated hepcidin levels. But low hepcidin levels in critically ill COVID-19 patients have also been reported in a study and this was in concomitance with hypoferremia [18]. In such cases, hypoferremia may be caused due to reasons other than hepcidin elevation. For example, this could be due to the putative hepcidin-mimetic action of the viral protein [81], which would block ferroportin and thereby reduce iron entry into the circulation leading to hypoferremia. Another reason could be elevated serum ferritin. In addition to elevating serum hepcidin, inflammation elevates ferritin levels, as observed in COVID-19 (Table 1). Ferritin can sequester a large number of iron ions, and the higher affinity of iron to ferritin and lactoferrin than transferrin (although transferrin levels are reduced in COVID-19) may assist in this sequestration process and contribute to hypoferremia. Interestingly, intracellular iron accumulation during inflammation may also increase intracellular ferritin [38], further locking the iron inside cells. In addition, increased levels of serum lipocalin-2 during COVID-19 (Table 1) may scavenge iron and support the development of hypoferremia. As such, this is not a COVID-19-specific response, lipocalin-2 generally supports mucosal, cellular and systemic hypoferremia during inflammation [97].

### What are the systemic consequences of inflammation-induced hypoferremia in COVID-19? Anemia of inflammation

The world health organization defines anemia as a condition wherein there is low hemoglobin concentration or low red blood cell count compared to the norm.

Inflammation-induced hypoferremia, as observed in COVID-19 causes iron-restricted erythropoiesis in the bone marrow [112], and reduces iron availability for the developing RBCs. Hypoferremia is reflected as low TSAT (Table 1), and accompanied by low transferrin levels (Table 1); the latter occurs due to inflammation [53]. Together, these events further aggravate iron unavailability to the developing RBCs, which may partly explain the low hemoglobin levels observed in some COVID-19 patients (Table 1).

Hemoglobin is the main oxygen transporting molecule, and so its level is one of the main determinants of the oxygen-carrying capacity of blood. Low hemoglobin level in COVID-19 patients, particularly those with pre-existing health complications can majorly contribute to tissue hypoxia [16]. As such, anemia limits oxygen delivery to tissues, thereby contributing to multi-organ failure in COVID-19 patients [16]. Thus, inflammation-induced hypoferremia forms the basis for anemia of inflammation in COVID-19.

Typically, anemia of inflammation occurs during inflammatory body states and is characterized by low serum iron levels despite adequate iron stores (i.e., normal, or high ferritin). All these three scenarios (inflammation, low serum iron and ample iron stores) are observed in COVID-19 (Table 1). Anemic COVID-19 patients show the presence of high inflammatory markers like IL6 and CRP [12]. Thus, lack of iron availability to the developing RBCs (due to inflammation) despite elevated serum ferritin, high ferritin deposition in the liver and high intracellular iron sequestration in cells leads to anemia of inflammation in COVID-19 patients. Elevated serum hepcidin is the main reason for this. Moreover, in anemia of inflammation, there is suppression of erythrocyte production because of the action of cytokines on erythroid progenitors [121]. Thus, the inflammation caused by COVID-19 can reduce both serum iron levels and erythrocyte production.

Absolute iron deficiency is a condition wherein there are no or low iron stores in the liver, spleen, and bone marrow. Functional iron deficiency is when iron stores are sufficient or elevated, but iron is not available for incorporation in the developing erythrocytes for erythropoiesis. The latter occurs due to high levels of systemic hepcidin [122]. Accordingly, hypoferremia (low serum iron) in the absence of elevated hepcidin is likely to reflect absolute iron deficiency. However, if hypoferremia is used as a sole parameter (without considering hepcidin) to assess body iron status, then it is not possible to differentiate between absolute and functional iron deficiency. The determination of serum hepcidin levels is very important for this purpose.

### Interferons may aggravate COVID-19-related anemia

The cytokine storm in COVID-19 not only reflects excessive production of cytokines (which can eventually cause multi-organ damage and death), but also shows imbalanced levels of types I, II, and III interferons. Generally, the interferons (IFNs) are produced in response to viral infections and other pathogens. These facilitate proper functionality of the immune system. IFN-γ helps in the differentiation of cytotoxic T cells. These T cells produce cytokines that restrict viral replication and kill virally-infected cells [123]. Reduced serum IFN-γ level has been identified as a risk factor of lung fibrosis in COVID-19 [124].

However, IFN-γ can play a role in COVID-19-associated anemia. Mice models have shown that IFN-γ inhibits erythropoiesis and reduces the life span of erythrocytes, thereby causing anemia [125]. Also, IFN-γ (along with IL6 and TNF-α) promotes iron uptake by the macrophages [126], thereby restricting iron availability to the developing erythrocytes. Additionally, along with IL1 and TNF-α, IFN-γ inhibits erythropoietin production in the kidney, which hinders erythroid-progenitor-cells’ differentiation and proliferation [21]. Moreover, inflammation-related mechanisms can cause hepatic and splenic macrophages to upregulate erythrophagocytosis and decrease erythrocyte half-life and erythrocyte numbers [119].

IFN-γ levels are higher in the early stages of COVID-19 infection, higher in non-survivors than survivors, and it is an independent risk factor associated with COVID-19-related mortality [127]. Thus, elevated IFN-γ levels may play a role in aggravating COVID-associated anemia.

Collectively, the data indicates a dual role of IFN-γ in COVID-19 whereby it has the potential of both, providing protection and aggravating disease severity. Usage of a regulated dosage of IFN-γ for ameliorating COVID-19 symptoms depending on disease stage/severity can be envisaged.

### Proportion of anemic patients

Pre-existing anemia in COVID-19 patients is associated with an elevated risk for in-hospital mortality. A study showed that the prevalence of anemia in COVID-19 patients (as measured by the drop in hemoglobin levels) increased from 44% to approximately 88% by 2 weeks of hospitalization [21]. In another study, about 25% of hospitalized COVID-19 patients were anemic at the time of admission and most patients showed anemia of inflammation. Functional iron deficiency was observed in 80% of patients upon hospital admission. This was associated with advanced inflammation and longer stay in hospital [49]. Another study showed that two months after COVID-19 onset, 30% patients still showed iron deficiency and 9% showed anemia. These anemic patients mostly showed anemia of inflammation, while some others showed iron deficiency anemia [12]. In a study conducted in Iran, about 48% of hospitalized COVID-19 patients (mean age 64.43 ± 17.16 years) were anemic. Several factors were associated with anemia such as sex, age, BMI and higher frequency of cardiovascular and kidney diseases, diabetes, hypertension and cancer [85]. Anemia is more prevalent in the elderly [128], and they may be less likely to be treated in ICU because of poor outcomes related to age [129].

### Autoimmune hemolytic anemia and COVID-19

Autoimmune hemolytic anemia (AIHA) is acquired hemolysis wherein the host’s immune system attacks the host’s red blood cells antigens. AIHA or suspected AIHA in the setting of COVID-19-induced hyperinflammation has been reported in several studies [130–133]. Hemolytic anemia in COVID-19 appears to be multifactorial. One proposal is that COVID-19-induced hyperinflammation alters antigen presentation, which creates cryptic antigens. These stimulate T cells, which in turn stimulate autoreactive B cells to generate antibodies against the cryptic antigens. The generated antibodies coat the red blood cells leading to a positive direct antiglobulin test in approximately 45% of COVID-19 patients [130].

### Hypoxemia, iron, and COVID-19

In COVID-19, infection and inflammation cause lung dysfunctionality resulting in low oxygen supply to the blood. The condition of low levels of oxygen in blood is referred to as hypoxemia. It can cause severe damage to all body organs. The severity of hypoxemia is independently associated with in-hospital mortality, and it is a predictor of ICU admission in COVID-19 patients [134]. Interestingly, patients with severe hypoxemia have shown significantly lower level of serum iron than those with non-severe hypoxemia and a tendency of lower serum transferrin and TSAT than patients with non-severe hypoxemia [17]. While this links iron and iron transport mechanisms with hypoxemia, the consequence of this combination (hypoxemia together with low iron availability for the developing RBCs) worsens COVID-19 pathology.

## Summary

Reported iron-related alterations in the sera of COVID-19 include decrements in iron, transferrin, transferrin saturation and hemoglobin (can show normal levels), and increments in ferritin, hepcidin (can be low), soluble transferrin receptor (in ICU patients) and lipocalin-2. Serum levels of iron and iron-related proteins can be used for COVID-19 prognosis. Membrane-bound transferrin receptor may facilitate viral entry. So, it is a potential target for antiviral therapy. Lactoferrin can provide natural defense by preventing viral entry and/or inhibiting viral replication.

Anemia in COVID-19 is primarily due to heightened inflammation (anemia of inflammation). It may be caused due to a combination of events such as i) cytokine-induced elevation in hepcidin that inhibits intestinal iron absorption and traps iron within iron-storing cells, ii) action of interferons, iii) increment in ferritin expression that occurs due to increased intracellular iron as well as inflammation, and iv) inflammation-induced reductions in transferrin and erythrocyte production. There could be pre-existing anemia/iron deficiency in COVID-19 patients due to gastrointestinal or urinogenital bleeding. Anemia could occur or could be aggravated due to iatrogenic blood draws.

## Data Availability

Not applicable.
